# WHONET: an information system for monitoring antimicrobial resistance.

**DOI:** 10.3201/eid0102.950209

**Published:** 1995

**Authors:** T F O'Brien, J M Stelling

**Affiliations:** whonet@bustoff.bwh.harvard.edu


### WHONET: An Information System for Monitoring Antimicrobial Resistance

WHONET is an information system developed to support The World Health Organization's (WHO) goal of global surveillance of bacterial resistance to antimicrobial agents. Microbiologists, clinicians and infection control workers may use its software to enhance monitoring of drug resistance in their hospitals and communities and to merge their files into national, regional, and global networks for surveillance of drug resistance. WHONET software can be installed on personal computers and be configured for the locations of the patients a laboratory serves and for the antimicrobial agents it tests. The program accepts susceptibility test results and allows printing of reports and logbooks and retrieval of data. If the laboratory already has a computerized reporting system, a translation program can be created to download the laboratory's files into WHONET. Either way, the microbiologists and other infectious disease specialists gain new analytical tools to monitor and manage susceptibility test quality and the spread of drug resistance locally and outside their area.

WHONET can also analyze stored data. From a single screen, a WHONET user selects the type of analysis to run, the species of bacteria to analyze, the subsets of isolates to include (e.g., all, isolates from urine only, and isolates resistant to gentamicin and from certain locations), and the antimicrobial agents and period to examine. Types of analyses include percentage of data categorized as resistant, intermediate, or susceptible by standard or other breakpoints; distributions of test measurements (zone diameter, minimal inhibitory concentration) in the form of histograms; scatterplots comparing measurements for different agents or methods for the same isolates; and line listings of isolates grouped by combinations of agents to which they are resistant (antibiotypes) to trace distinctive strains. Isolates with uncommon antibiotypes can also be flagged on entry so that they may be rechecked while still available, and local outbreaks can be detected early.

Although test results are entered and monitored locally on software configured for local use, they are filed in a universal file format so that any copy of the program can analyze the files of any laboratory. This feature has enabled groups of users in 10 countries to set up passive surveillance systems by pooling and analyzing their files collaboratively. WHONET assists such initiatives by providing file encryption options to ensure confidentiality before data are pooled and analyzed.

Ongoing local analysis by local workers is the foundation of the system. It detects local problems in testing, which no laboratory can avoid entirely, and thus improves the overall quality of the files. It delineates local spread of drug-resistant strains, which aids infection control and can explain and correct uncommon prevalence of certain types of drug resistance at certain sites. It allows local workers to distinguish their problems from those of other sites and focus on infection control or antimicrobial use that might be related to those problems.

Expansion of the system has been recommended by the WHO Scientific Working Group on Monitoring and Management of Bacterial Resistance to Antimicrobial Agents. For more information or for participation contact

**Thomas F. O'Brien and John M. Stelling**
WHO Collaborating Center for Surveillance of
Resistance to Antimicrobial Agents
Microbiology, Brigham and Women's Hospital
Boston, MA 02115, USA
tel (1-617) 732-6803
fax (1-617) 732-4144
Internet: whonet@bustoff.bwh.harvard.edu

### Recommendations for Preventing the Spread of Vancomycin Resistance

CDC's Hospital Infection Control Practices Advisory Committee (HICPAC) has published "Recommendations for Preventing the Spread of Vancomycin Resistance." The recommendations focus on vancomycin-resistant enterococci (VRE).

The reported incidence of infection and colonization with VRE in U.S. hospitals has increased rapidly in the last 5 years. This increase has compounded the need for antimicrobial drugs to treat VRE infections. Most VRE are also resistant to multiple other drugs (e.g., aminoglycoside and ampicillin), which have been used for treating VRE infections. In addition, the possibility that the vancomycin-resistance genes present in VRE may be transferred to other gram-positive microorganisms, especially *Staphylococcus aureus*, is a serious public health concern.

Although the epidemiology of VRE has not been fully elucidated, and most enterococcal infections have been attributed to the patient's endogenous flora, recent studies have demonstrated that enterococci, including VRE, can be spread directly from patient to patient or indirectly by transient carriage on the hands of personnel or contaminated environmental surfaces and patient-care equipment.

In its recommendations, HICPAC stresses that the prevention and control of vancomycin resistance will require a coordinated, concerted effort from various departments of a hospital. Because the recommendations were developed with limited data and further research is needed to find cost-effective ways to control the spread of vancomycin resistance, HICPAC strongly encourages hospitals to develop their own institution-specific plans, which should stress the following elements: 1) prudent vancomycin use by clinicians, 2) education of hospital staff regarding vancomycin resistance, 3) early detection and prompt reporting of vancomycin resistance in enterococci and other gram-positive microorganisms by the hospital microbiology laboratory, and 4) immediate implementation of appropriate infection-control measures to prevent person-to-person transmission of VRE.

The recommendations were developed by HICPAC's Subcommittee on the Prevention and Control of Antimicrobial-Resistant Microorganisms in Hospitals and subject-matter experts and representatives of the American Hospital Association, American Society for Microbiology, Association for Professionals in Infection Control and Epidemiology, Infectious Diseases Society of America, Society for Healthcare Epidemiology of America, and Surgical Infection Society. The recommendations were published in February in *Infection Control and Hospital Epidemiology* 1995;16:105-13 and will also be published in the April 1995 issue of the *American Journal for Infection Control.*

**Hospital Infection Control Practices Advisory Committee**
National Center for Infectious Diseases
Centers for Disease Control and Prevention
Atlanta, Georgia, USA

### Waterborne Cryptosporidiosis Threat Addressed

*Cryptosporidium parvum* was first recognized as a cause of human illness in 1976. From 1976 to 1982, the disease was reported rarely in the United States, primarily among the immunocompromised. In 1982, the number of reported cases began to increase dramatically along with the number of HIV-infected persons; outbreaks among immunocompetent populations also were reported. Recent municipal waterborne outbreaks of cryptosporidiosis in Texas (1984), Georgia (1987), and Oregon (1992), and a massive outbreak in Wisconsin in 1993 that affected more than 400,000 persons have raised awareness about the waterborne transmission of cryptosporidiosis. Since 1993, several smaller cryptosporidiosis outbreaks were reported in the United States: two were related to drinking water, six were linked to recreational water, and one was foodborne.

Cryptosporidiosis is caused by ingestion of the environmentally tough oocysts of the protozoan parasite *C. parvum*, an intracellular organism that can replicate in the gut epithelial cells of most mammals. Its oocyst is extremely resistant to chlorine, which is commonly used to treat municipal water.

In healthy persons, the disease lasts 1 to 2 weeks and can have considerable economic impact through absenteeism of those affected. In the immunocompromised, the disease is often severe, lifelong, and life-threatening. No effective therapy is available.

The magnitude of the 1993 Wisconsin outbreak and its association with a municipal water plant operating within existing state and federal regulations underlined the need for improved surveillance and coordination among public health agencies and spurred efforts for regulatory standards for *Cryptosporidium* in drinking water. During 1995-1996, the U.S. Environmental Protection Agency (EPA) intends to implement the Information Collection Rule, which requires utilities that serve populations of 100,000 or more and use surface water (lakes, rivers, streams) to test that water routinely for *Cryptosporidium* oocysts. If oocysts are found, the utility may also have to test finished water (tap water). Utilities that serve populations of 10,000 to 99,000 will also have to test source water, but for a shorter period. They will not be required to test tap water, even if oocysts are found. Authority to issue boil water advisories if oocysts are found varies from state to state. The health risks from ingesting low levels of *Cryptosporidium* are unknown. More than 300 representatives from 40 states and more than 25 regulatory, public health, water utility, and advocacy groups met at the Centers for Disease Control and Prevention (CDC) in Atlanta in September 1994 to discuss the prevention and control of waterborne cryptosporidiosis. Recommendations from the CDC workshop will be published in the next 2 to 3 months.

CDC held the first meeting of the Working Group on Waterborne Cryptosporidiosis in November 1994. The working group convenes biweekly by teleconference. For more information about the group, contact Margaret Hurd (phone: 404-488-7769, fax: 404-488-7761).

The working group has three main purposes: 1) promote a regular exchange of ideas, goals, activities, and proposals among individual scientists, agencies, and organizations interested in waterborne cryptosporidiosis; 2) make decisions on public health issues related to waterborne cryptosporidiosis; and 3) assemble smaller, more focused, task forces with expertise to develop, implement, and evaluate projects of the working group.

The working group has created task forces to assist local, state, and national public health departments, water utilities, and regulatory agencies in preparing for and managing outbreaks. The task forces have the following responsibilities:

